# Non-electrocardiography- and electrocardiography-gated computed tomography angiography for the evaluation of feline coronary arteries

**DOI:** 10.3389/fvets.2022.952412

**Published:** 2022-08-03

**Authors:** Junyoung Kim, Dae-Hyun Kim, Kitae Kim, Dayoung Oh, Junghee Yoon

**Affiliations:** ^1^Helix Animal Medical Center, Seoul, South Korea; ^2^College of Veterinary Medicine and the Research Institute for Veterinary Science, Seoul National University, Seoul, South Korea; ^3^College of Veterinary Medicine, Chungnam National University, Daejeon, South Korea; ^4^BIEN Animal Medical Center, Bucheon, South Korea

**Keywords:** cardiomyopathy, circumflex, paraconal branch, septal branch, vertebral heart score

## Abstract

**Objectives:**

Few studies have directly compared the clinical feasibility of electrocardiography-gated and non-electrocardiography-gated multidetector computed tomography for evaluating coronary arteries in veterinary medicine. We aimed to characterize and visualize feline coronary arteries using these two imaging modalities. We hypothesed that ECG-gated MDCT is superior to or advantageous to the non-ECG gated.

**Methods:**

This prospective, controlled, comparative pilot study examined six client-owned cats (five clinically normal and one with hypertrophic cardiomyopathy) using non-electrocardiography-gated and retrospective electrocardiography-gated scans. Optimal non-electrocardiography scan timing or electrocardiography-gated R-R reconstruction interval for coronary artery visualization was determined. The degree of opacification and sharpness of proximal coronary branches was subjectively graded; coronary dominance, left coronary artery branching types, and the diameter and length of coronary artery branches were also assessed.

**Results:**

Non-electrocardiography-gated images provided the least information on the bilateral coronary artery ostium and proximal segments, while electrocardiography-gated images clarified the detailed course of the main coronary branches at diastole in all cats. The degree of opacification and sharpness of the coronary arteries was subjectively evaluated as good/excellent in all cats. Coronary dominance (left: four; right: two) and left coronary artery branching types (three different patterns, two additional tortuous branches) varied. Body weight and sex were not significantly associated with coronary artery length or diameter. Vertebral heart score positively correlated with the right coronary artery and negatively correlated with the left main coronary artery.

**Clinical significance:**

Electrocardiography-gated multidetector computed tomography provides images with adequate resolution to identify the anatomy of feline coronary arteries. Detailed morphological knowledge of feline coronary vessels will enable novel diagnostic and therapeutic methods in veterinary medicine.

## Introduction

Multidetector computed tomographic (MDCT) angiography is considered the gold standard for evaluating coronary vessels in humans, permitting multiple interventional cardiologic procedures (1–4). Although coronary artery (CA) stenosis, reported commonly in human medicine, rarely occurs in animals, various congenital or acquired coronary diseases have been reported in veterinary medicine (4–8). Anomalous coronary arteries (CAs) in combination with pulmonic stenosis have been reported predominantly in dogs (4, 5, 8). In cats, a morphological alteration has been observed that has also been reported in the long-tailed chinchilla, where the right coronary artery is usually missing and only a single coronary artery exists (9). Such knowledge may be especially helpful in explaining the pathophysiology of CA diseases (9). Therefore, understanding the anatomy, terminology, and clinical implications of these CA anomalies is critical for the diagnosis and treatment of veterinary patients.

Electrocardiography (ECG)-gated MDCT links image acquisition to specific phases of the cardiac cycle (10). In prospective ECG-gated scans, image acquisition is only triggered during a certain phase of the cardiac cycle, commonly at the end-diastolic phase, during which the heart is mostly motionless (10). This is favored for morphological evaluations, as adequate image quality is provided at a lower radiation dose relative to non-ECG and retrospective ECG-gated scans. However, prospective scans do not acquire a full-cycle dataset; further, in patients with irregular heart rhythms or higher heart rates, prospective scans may lead to the acquisition of an incomplete dataset and may fail to capture the optimal phase of the cardiac cycle during diastole. In retrospective scans, image acquisition is triggered over the entire length of one or more cardiac cycles, and the dataset can be retrospectively separated into different phases of the cardiac cycle (usually in 5–10% intervals). Thus, cardiac function and morphology can be evaluated in different phases of the cardiac cycle, and images can depict the beating heart over the entire cardiac cycle (10).

Previous reports on canines indicated that ECG-gated MDCT was more effective in identifying anomalous CAs than non-ECG-gated MDCT, in terms of imaging the cardiac morphology and the detailed CA courses, due to minimizing cardiac motion artifacts (4, 11–13). Although ECG-gated CT has the advantage of being able to control cardiac motion artifacts, animals have faster heart rates than humans, and beta-blockers exhibit limited efficacy in reducing heart rates in animals. A recent study successfully diagnosed CA anomalies using non-ECG, high-slice MDCT scans (14). Therefore, it is essential to consider the necessity and usefulness of non-ECG-gated CT imaging (5). However, there is a paucity of research directly comparing the clinical feasibility of ECG-gated and non-ECG-gated MDCT for evaluating CAs in veterinary medicine (5, 11, 12, 15). Therefore, we aimed to characterize and visualize feline CAs using non-ECG-gated and ECG-gated MDCT to describe the CA variations and to evaluate the features and clinical feasibility of non-ECG and ECG-gated MDCT in cats. We hypothesized that ECG-gated MDCT is superior to or advantageous to the non-ECG gated.

## Materials and methods

### Animals

This study was a prospective, controlled, comparative pilot study including cats that had been brought to an animal medical center for a complete medical check-up. The study design and care, as well as animal maintenance, followed protocols approved by the institutional animal care and use committee of Seoul National University (approval number: SNU-220113-4). Medical history and informed consent were obtained for all client-owned cats prior to study procedures. **Six** domestic short-haired cats with no clinical signs provided by the owners were included. Before the MDCT examination, all cats underwent basic health tests, including physical examination, complete blood counts, serum biochemistry, and electrolyte tests. N-terminal Pro-B-type natriuretic peptide (NT-proBNP) testing, thoracic radiography, and transthoracic echocardiography (Aplio 500, Toshiba, Canon Medical Systems Co., Otawara, Japan) were performed for cardiac evaluation. Using thoracic radiography, the vertebral heart score (VHS) was calculated. Two-dimensional, M-mode, and Doppler ECG examinations were performed on all cats. The time interval between all basic health tests and MDCT examinations for individual cats was within five days.

### Anesthesia

An intravenous 24-G catheter was placed in the right cephalic vein for premedication and contrast agent injection during MDCT. The cats were premedicated with butorphanol (0.2 mg/kg intravenously; 1 mg/mL, Butophan® Myungmoon Pharm Co., Ltd., Seoul, Republic of Korea), and general anesthesia induced with propofol (6 mg/kg intravenously; 10 mg/mL, Provive® 1%, Myungmoon Pharm Co., Ltd., Seoul, Republic of Korea), and maintained with isoflurane (Isotroy® 100, Troikaa Pharm Ltd., Gujarat, India) in a gas mixture of 100% oxygen in the air *via* an endotracheal tube. End-tidal carbon dioxide levels were maintained between 35 and 45 mmHg using a mechanical ventilator. Heart rate, oxygen saturation, and end-tidal carbon dioxide were continuously monitored during anesthesia *via* ECG and pulse oximetry. Data acquisition was initiated within 5–10 min after anesthesia induction to ensure the stability of anesthetic conditions. For individual scans, apnea was induced by breath-holding at inspiration immediately before the scan. All cats were monitored until recovery from anesthesia. The total time from the start to the end of anesthesia was recorded.

### Non-ECG-gated and ECG-gated MDCT

**Figure 1:**
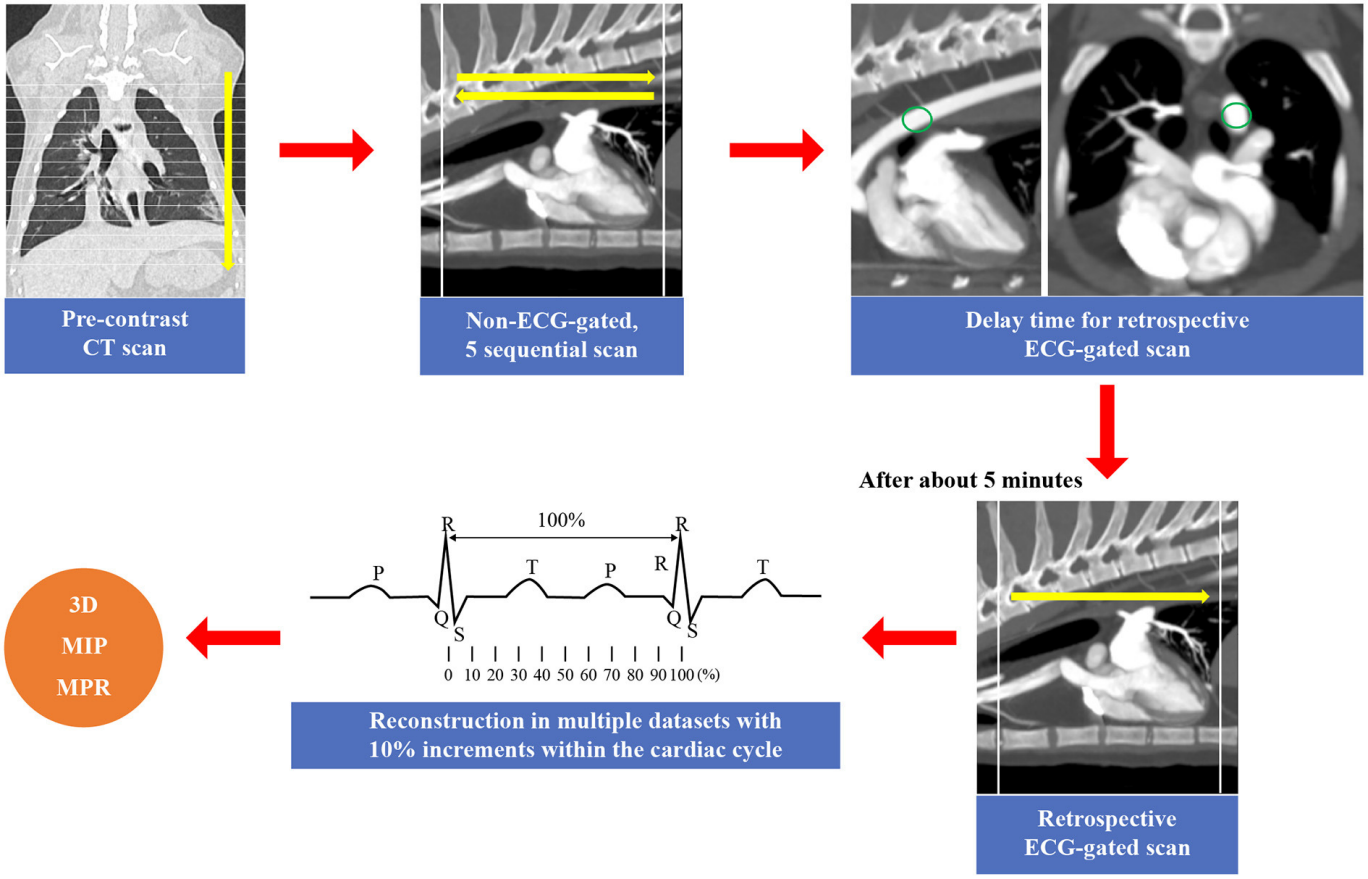
The scan method of the cardiac computed tomography (CT) in this study. In this study, all cats were examined using non-electrocardiography (ECG)-gated scans, followed by ECG-gated scans. First, a pre-contrast CT scan of the full thorax from the thoracic inlet to the caudal-most border of the lungs was performed prior to the post-contrast studies. For non-ECG-gated scans, five sequential scans, cranial to caudal and vice versa, from the second rib to the cranial border of the diaphragm, were performed at 5-s intervals (14). The delay time for retrospective ECG-gated scans was determined based on non-ECG-gated sequential images. After that, retrospective ECG-gated scan was performed (10). For data postprocessing, images were reconstructed in multiple datasets, with the temporal reconstruction window increasing in 10% increments within the cardiac cycle, centered over the 0–90% R-R interval. Maximum intensity projection (MIP), three-dimensional (3D) volume-rendered, and multiplanar reconstructions (MPR) were applied as needed.

MDCT CA angiography was performed using an 80-row, 160-multislice CT system (Aquilion Lightning, Canon Medical Systems Co., Otawara, Japan). For both non-ECG-gated and ECG-gated MDCT, cats were positioned in sternal recumbency on a CT table with the neck extended and the forelimbs positioned caudally. ECG leads were attached to the paws; ECG data were recorded simultaneously during spiral MDCT examination. Scan parameters were as follows: voltage, 120 kVp (kVp: kilovoltage peak); gantry speed, 0.5 s/rotation; slice collimation, 0.5 mm × 80; 150 mA; 0.5 mm slice thickness; and pitch factor, 0.813. All cats were examined using non-ECG-gated scans, followed by ECG-gated scans after a short interval (5 min) to wash out the contrast medium from the heart. In all cats, a pre-contrast MDCT scan of the full thorax from the thoracic inlet to the caudal-most border of the lungs was performed prior to the post-contrast studies. For non-ECG-gated scans, a biphasic injection was administered, comprising a non-ionic contrast medium (1.5 mL/kg; 300 mg/I/mL, Omnipaque® GE Healthcare, Seoul, Republic of Korea), followed by a saline flush (1.5 mL/kg) into the cephalic vein using a dual power injector (OptiVantageTM DH, Mallinckrodt, Dublin, Ireland) at a rate of 1.5 mL/s. Image acquisition began 8 s after the initiation of the contrast injection. Subsequently, five sequential scans, cranial to caudal and vice versa, from the second rib to the cranial border of the diaphragm, were performed at 5-s intervals. To reduce radiation exposure and anesthesia time, the delay time for retrospective ECG-gated scans was determined based on non-ECG-gated sequential images. Contrast medium administration for ECG-gated MDCT was conducted in the same manner as that for non-ECG-gated scans. For data postprocessing, images were reconstructed in multiple datasets, with the temporal reconstruction window increasing in 10% increments within the cardiac cycle, centered over the 0–90% R-R interval. All images were reviewed by three veterinary diagnostic imaging experts on a dedicated viewing station using specialized software (Vitrea 7.12, Vital Images, Minnetonka, MN, USA). All images obtained in the non-ECG-gated and ECG-gated MDCT examinations were evaluated, and the scan timing and phase with the least motion were selected for image reconstruction in standard tomographic views. Maximum intensity projection, three-dimensional volume-rendered, multiplanar, and curved reconstructions were applied as needed to optimize CA visualization (Figure 1).

### Criteria for CA analysis

**Figure 2:**
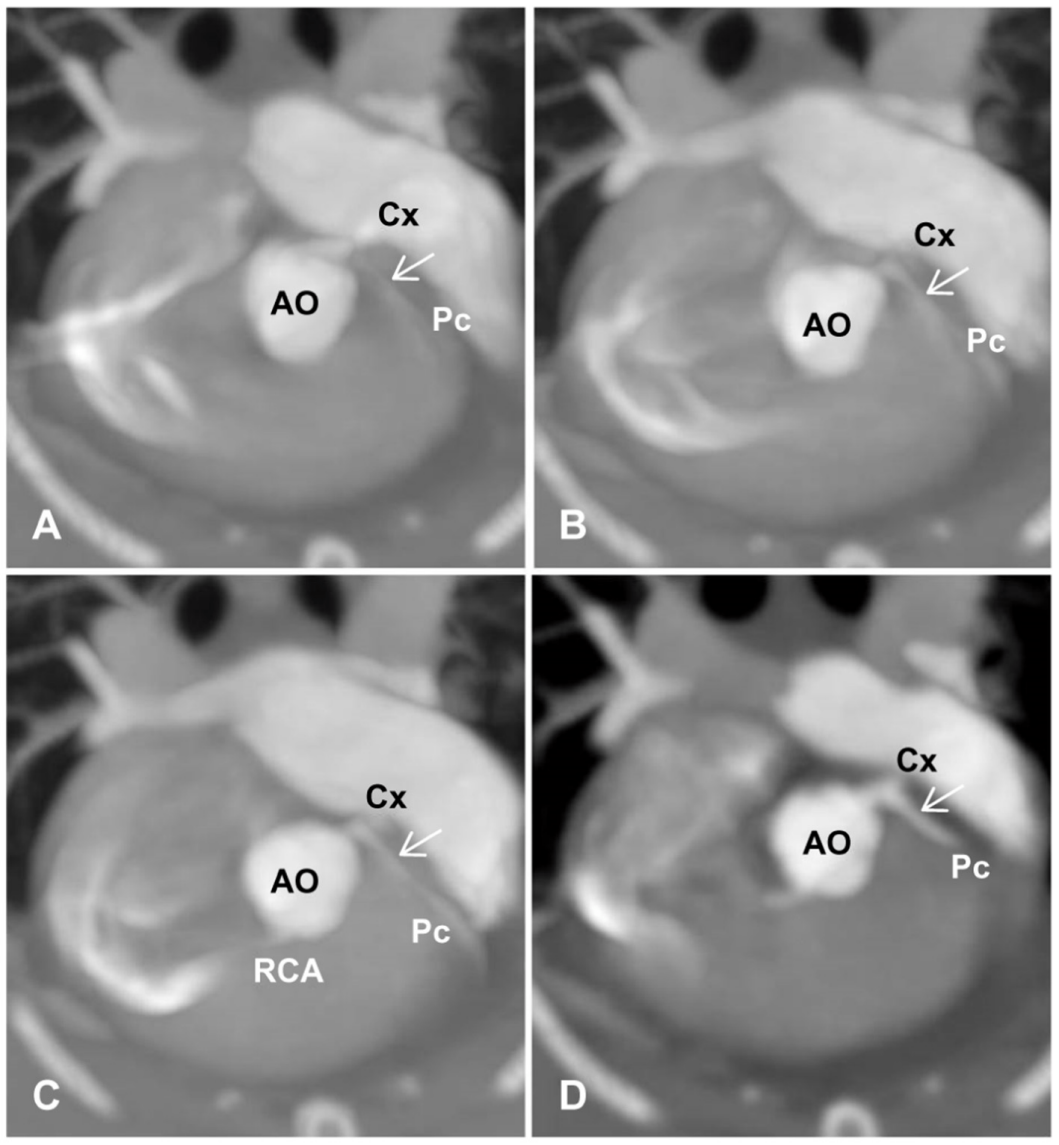
Degree and sharpness of feline coronary arteries (arrows). **(A)** 0 (poor): poor opacification of the coronary branches with severe blurring prohibiting the evaluation of coronary arteries at their origins. **(B)** 1 (mild): origins of coronary arteries with adequate opacification can be evaluated but are non-diagnostic for evaluating the course of coronary arteries with moderate blurring. **(C)** 2 (good): adequate opacification and sharpness of coronary branches with an acceptable degree of blurring, where mild artifacts may be present, but origins of coronary arteries and distal branches can be evaluated. **(D)** 3 (excellent): excellent opacification and sharpness of coronary branches with minimal artifacts. AO, aorta; Cx, circumflex; Pc, paraconal interventricular; RCA, right coronary artery.

**Figure 3:**
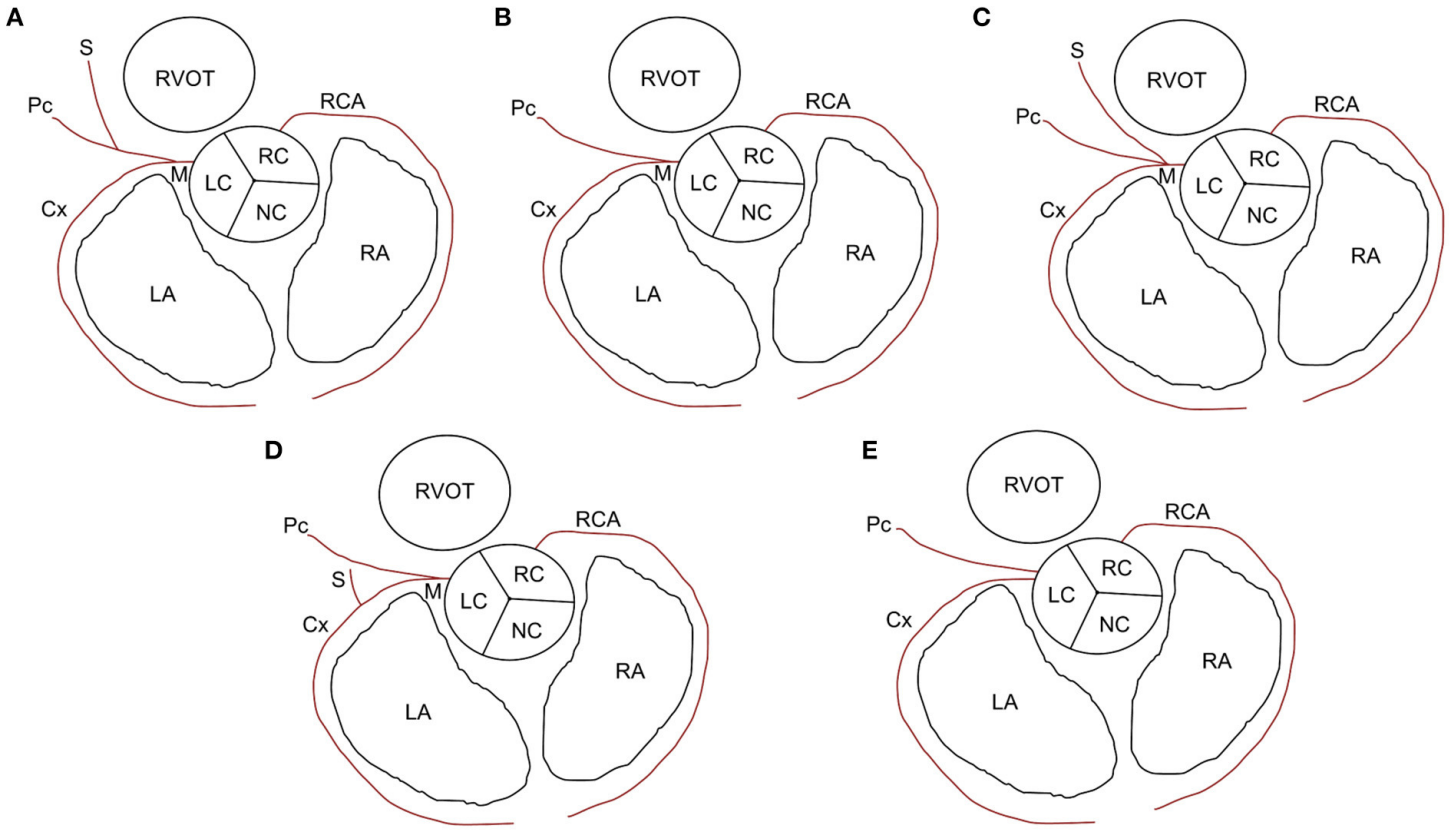
Types of left coronary artery branching in cats. **(A)** Type I: short left main coronary artery (M) giving rise to the circumflex (Cx) branch and paraconal interventricular (Pc) branch, which branches off to the septal (S) branch. **(B)** Type II: M gives rise to the Cx and Pc branches without an S branch. **(C)** Type III: M gives rise to the Cx, Pc, and S branches. **(D)** Type IV: M gives rise to the Pc branch and Cx branch, which branches off to the S branch. **(E)** Type V: no M and two separate ostia for Cx and Pc branches originating from the aorta. LA, left atrium; LC, left coronary cusp; NC, noncoronary cusp; RA, right atrium; RC, right coronary cusp; RCA, right coronary artery; RVOT, right ventricular outflow tract.

The non-ECG and ECG-gated images were assessed based on the following:

(a) Optimal scan timing or phase in the R-R reconstruction interval for the best visualization of the proximal CA branches (from the start point to ~3–5 mm).
(b) The degree of opacification and sharpness of the proximal CA branches, which were subjectively graded as poor (0), mild (1), good (2), or excellent (3) (Figure 2). The presence and types of artifacts in all images were described and recorded.
(c) Coronary dominance (right, left, or codominance), which was defined as a left CA (LCA) or right CA (RCA) extending beyond the crux cordis (intersection between the interatrial, subsinosus interventricular, and coronary sulci) and the origin of the subsinosal interventricular branch (16, 17).
(d) Classification of LCA branching into **five** main types of the LCA proximal segment, as previously described (Figure 3) (9). Type I branching was characterized by a double-branched left main coronary artery (LMCA), giving rise to the circumflex (Cx) and interventricular paraconal (Pc) branches, which branched off to the septal (S) branch. Type II was characterized by a double-branched LMCA, giving rise to the Cx and Pc branches without an S branch. Type III was characterized by a triple-branched LMCA, giving rise to the Cx, Pc, and S branches. Type IV was characterized by a double-branched LMCA, giving rise to the Pc and Cx, which branched off to the S branch. Type V was characterized by the lack of an LMCA and two separate ostia for the Cx and Pc originating from the aorta.
(e) The diameter and length of the LCA and RCA according to the segmentation of their branches, based on a method used in previous canine studies (5, 16). The maximum vessel diameter was measured at the origin of each coronary branch. The summed length of each left coronary branch, defined as total LCA herein, was then calculated.

### Statistical analyses

All data are expressed as the mean ± standard deviation (SD). Statistical analyses were performed using GraphPad Prism 9.0.2 software (GraphPad Software Inc., San Diego, CA, USA). Data were assessed for conformance to a normal distribution using the Shapiro-Wilk test. Linear correlations of body weight (BW), sex, and VHS with the diameter and length of the major coronary branches were identified using Pearson's chi-squared test. Pearson's correlation coefficients, 95% confidence intervals (CIs), and *P*-values were calculated for each contrast. The mean values of sex-related parameters were compared using a two-tailed unpaired Student's *t*-test. *P*-values < 0.05 were considered statistically significant.

## Results

### Animals and feasibility of MDCT angiography

Six cats (mean age, 4.85 ± 3.74 years; mean BW, 4.82 ± 1.0 kg; mean VHS, 7.03 ± 0.72 v) comprised three spayed females and three males (one intact and two neutered) (Table 1). Five cats exhibited normal results in all basic health tests. One cat (Case 6) was diagnosed with hypertrophic cardiomyopathy (HCM) and had a positive NT-proBNP test result (Table 1), with systemic blood pressure of 150 mmHg and normal serum thyroid hormone concentration of 2.1 μg/dL (reference range, 0.6–3.9 μg/dL).

**Table 1:** Signalment, vertebral heart score (VHS), and N-terminal Pro-B-type natriuretic peptide (NT-proBNP) results of the cats.

**Case No**.	**Age (years)**	**Sex**	**BW (kg)**	**VHS**	**NT-proBNP**
1	3	Spayed female	4.14	7.2 v	Negative
2	3.6	Intact male	4.24	6.0 v	Negative
3	1	Spayed female	4.4	7.2 v	Negative
4	2.7	Spayed female	4.48	6.9 v	Negative
5	8	Castrated male	6.8	6.7 v	Negative
6	10.8	Castrated male	4.85	8.2 v	Positive

BW, body weight.

MDCT angiography using non-ECG-gated and ECG-gated scans was performed successfully on all cats. The average total time from the induction of anesthesia to the conclusion of MDCT examination was 30 min (range, 25–35 min) per cat. Heart rates during the MDCT scan were 120–150 bpm in all cats. No complications associated with the anesthetic protocol were documented in any cat. The delay time for the retrospective ECG-gated scan, determined using the second scan timing images for non-ECG-gated scanning, was set at 14 s in all cats.

### Selection of optimal coronary images based on comparison of Non-ECG and ECG-gated images

**Figure 4:**
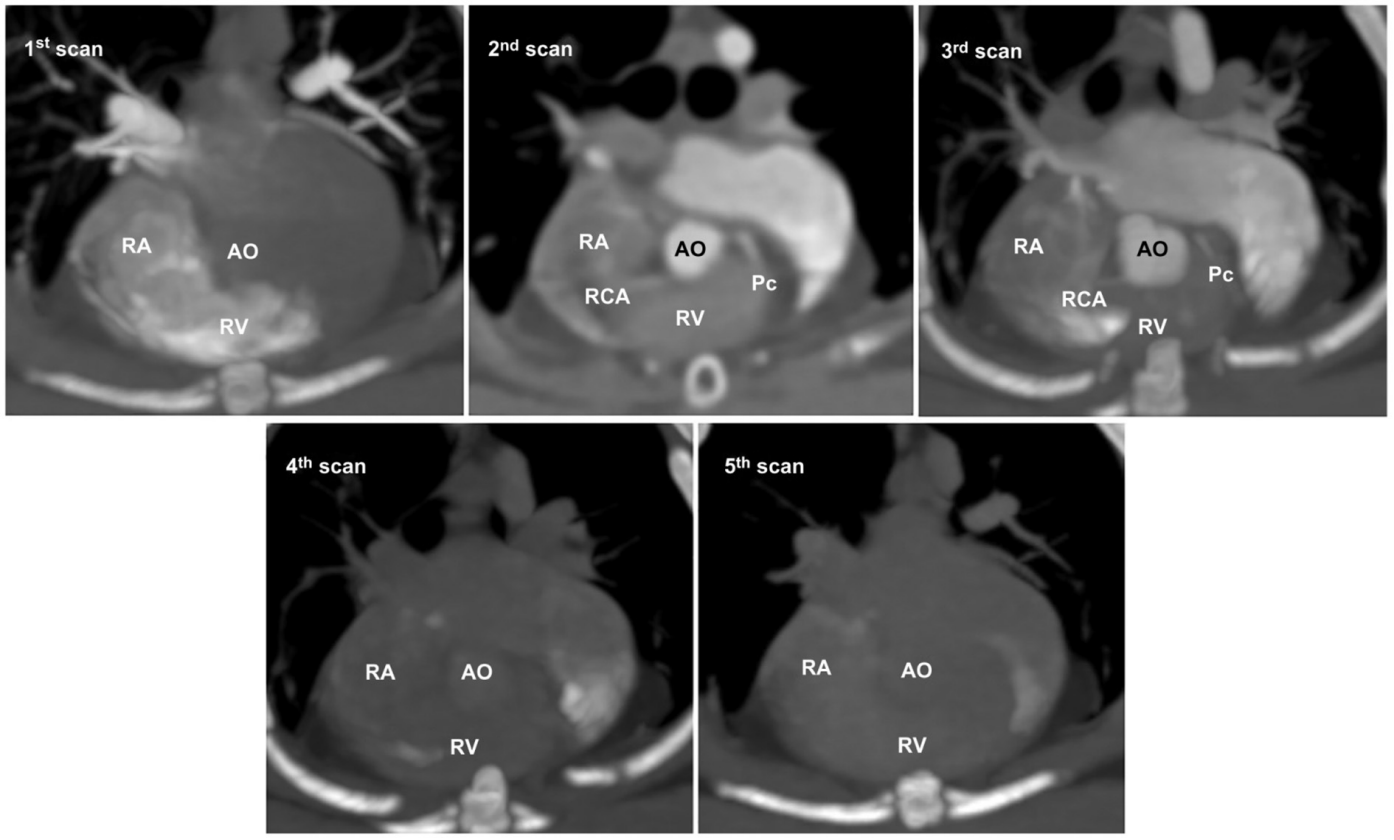
Five representative sequential non-electrocardiography (ECG)-gated multidetector computed tomographic (MDCT) images. In non-ECG-gated images, the first image shows no contrast enhancement in the left heart. The second and third images allow visualization of the left and right main coronary stems at the origin but produce non-diagnostic images for evaluating the detailed course of the coronary arteries owing to severe cardiac motion and blurring. The fourth and fifth images show poor opacification owing to the wash-out of the contrast medium. AO, aorta; Pc, paraconal interventricular branch; RA, right atrium; RCA, right coronary artery; RV, right ventricle.

In non-ECG-gated images, the first scan exhibited no contrast enhancement in the left heart, which precluded the evaluation of the left heart and CAs in all cats (Figure 4). The second and third scans permitted visualization of the left and right main coronary stems at their origins but produced non-diagnostic images owing to blurring, streaking, and stair-stepping caused by cardiac motion artifacts, which obscured a detailed evaluation of the course of the CAs (Figure 4). The fourth and fifth scans did not provide suitable images owing to poor opacification of the left heart and CA resulting from wash-out of the contrast medium (Figure 4).

In ECG-gated images, the optimal R-R reconstruction intervals for both the LCA and RCA were 70, 80, and 90% in two, one, and two cases, respectively (Table 2). In line with previous canine investigations, the CAs in all cats were best visualized during the late end-diastolic phase, when the coronary flow is expected to be maximal and cardiac motion is reduced. Common artifacts included blurring and stair-step appearance secondary to cardiac motion. These artifacts were more frequently observed in non-ECG-gated images than in ECG-gated images.

**Table 2:** Results of optimal R-R reconstruction intervals and degrees of opacification and sharpness of the proximal coronary arterial branches.

**Case No**.	**R-R interval (%)**	**Grade**
1	90	3
2	70	3
3	90	2
4	70	2
5	80	2
6	90	3

Grading system: 0, poor; 1, mild; 2, good; 3, excellent.

### Degree of opacification and sharpness of feline CA branches

In selected images, the degree of opacification and sharpness of the proximal coronary branches was subjectively evaluated as excellent (3/6; 50%) or good (3/6; 50%) (Table 2). ECG-gated images enabled the characterization of the course of coronary branches based on diagnostic images in all cats.

### Coronary dominance

Four cats exhibited left dominance; two exhibited right dominance (Table 3).

**Table 3:** Coronary dominance and branching types of the left coronary artery (LCA).

**Case No**.	**Coronary dominance**	**Branching types of the LCA**
1	Left	Type II
2	Left	Type III
3	Left	Type I
4	Right	Type II
5	Left	Type II
6	Right	Type II

Type I: a double-branched left main coronary artery (LMCA) that gives rise to the circumflex (Cx) and interventricular paraconal (Pc) branches, which further branches off and gives rise to the septal (S) branch.

Type II: a double-branched LMCA that gives rise to the Cx and Pc branches without an S branch.

Type III: a triple-branched LMCA that gives rise to the Cx, Pc, and S branches.

### LCA branching types

**Figure 5:**
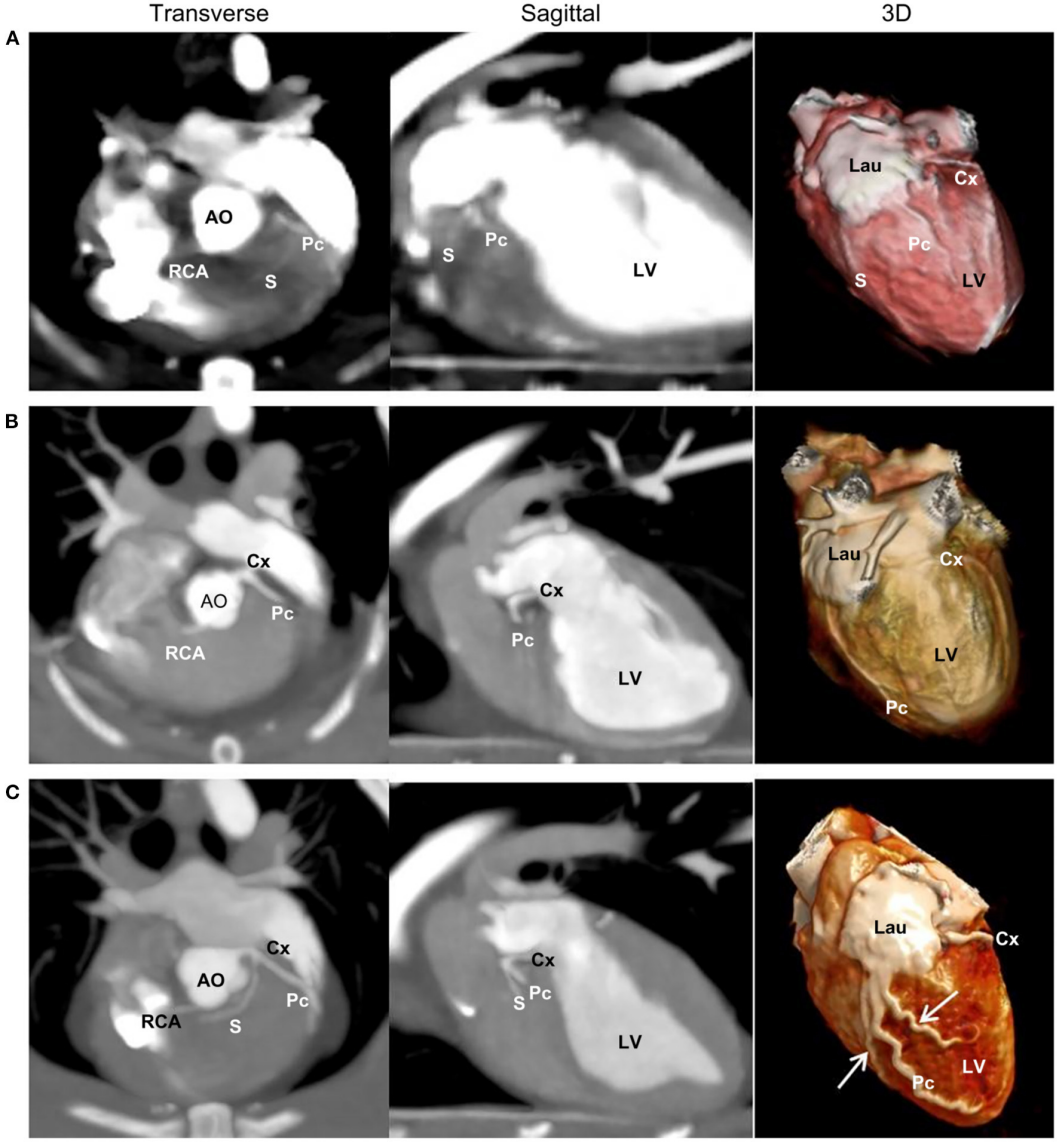
Two-dimensional transverse and sagittal views and three-dimensional (3D) multidetector computed tomographic images of the three left coronary branching types. Type I **(A)** is characterized by a short left main coronary artery (LMCA) giving rise to the circumflex (Cx) branch and paraconal interventricular (Pc) branch, which branches off to the septal (S) branch. Type II **(B)** is characterized by a short LMCA giving rise to the Cx and Pc branches without an S branch. Type III **(C)** is characterized by a short LMCA giving rise to the Cx, Pc, and S branches. **(C)** Also illustrates severe tortuosity of the Pc branches and two additional intermediate efferent branches (arrows) from the proximal Pc branch. AO, aorta; Lau, left auricle; LV, left ventricle; RCA, right coronary artery.

CA branching types were characterized as type II in four cats, type I in one cat, and type III in one cat (Table 3). In the type III case, the Pc exhibited severe tortuosity, with two additional intermediate efferent branches originating from the proximal Pc branch, which were arbitrarily termed intermediate interventricularis paraconalis branches (Figure 5).

### Segmentation of the CAs

**Figure 6:**
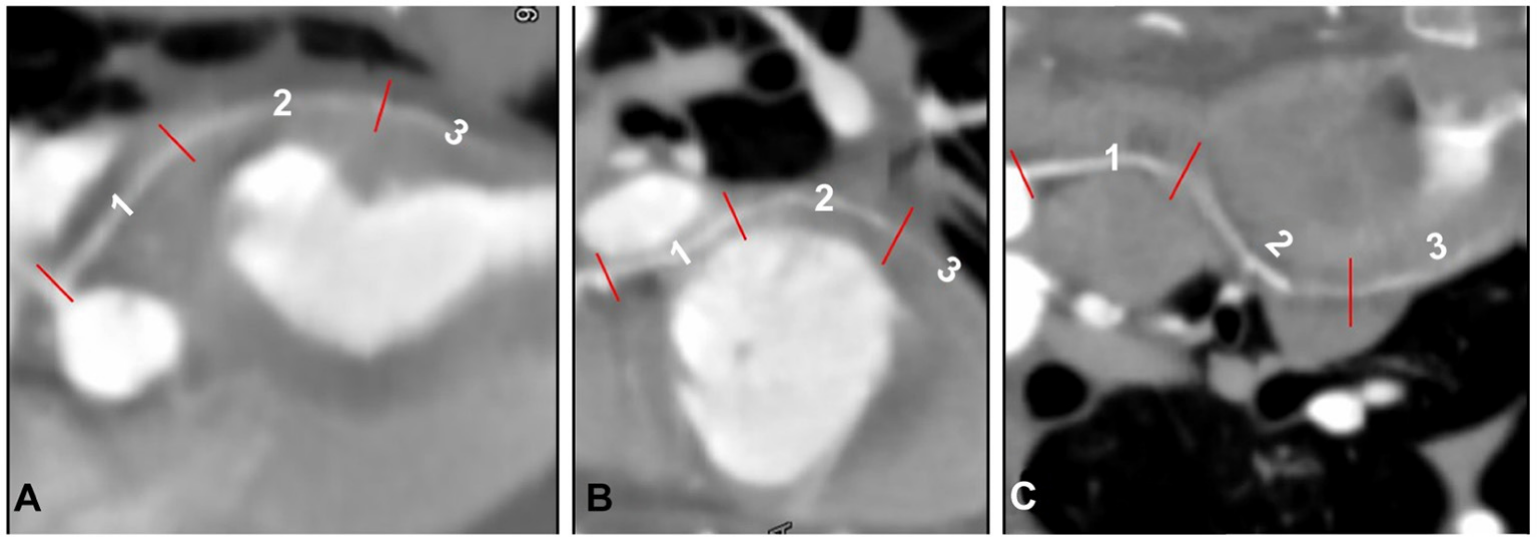
Segmentation of the paraconal interventricular (Pc) branch **(A)**, circumflex (Cx) branch **(B)**, and right coronary artery (RCA) **(C)** classified by adapting a previously described segmental coding system (16), displayed as curved multiplanar reconstruction.

Left and right CA branches were further classified by adapting a previously described segmental coding system, whereby an arterial portion located between two reference points was considered an angiographic segment (Figure 6) (16). The LCA originated from the left sinus of Valsalva in all cats. When the LMCA was present, it was classified as a single distinct segment before branching off, as classified above. The Cx and Pc were divided into three segments, as was the RCA (a total of 10 angiographic segments).

The three Cx segments were termed Cx1 (cranial segment), Cx2 (lateral segment), and Cx3 (caudal segment). The cranial segment ran caudally and ventrally to the left auricle and continued caudolaterally along the lateral surface of the left ventricular myocardium. The lateral segment ran perpendicular to the scanning plane into the coronary groove, laterally to the left atrioventricular junction, and ventral to the left circumflex vein. The caudal segment ran caudomedially to the left atrium, parallel to the scanning plane and along the caudodorsal surface of the left ventricle, and continued to run in the subsinosus interventricular groove, ending as the subsinosus interventricular branch. The Pc was classified into three segments: dorsal (Pc1), medium (Pc2), and apical (Pc3). The dorsal segment ran along the mid-dorsal aspect of the heart, caudoventrolaterally to the left, just caudal to the main pulmonary artery, ventromedially to the left auricle, and almost parallel to the scanning plane. The medium segment continued to run caudally along the mid-surface of the heart and within the Pc interventricular groove before ending as Pc3 along the ventral portion of the heart (apical portion). The S branch, which was observed only in **two** cats, ran laterally to the aortic bulb toward the interventricular septum (S1) and turned perpendicular to the scanning plane just ventral to the aortic bulb supplying the mid-caudal aspect of the interventricular septum (S2).

The RCA originated from the right sinus of Valsalva and was classified into three segments: RCA1, RCA2, and RCA3. The first segment ran ventrally in the coronary groove, between the main pulmonic trunk and right atrium, almost parallel to the scanning plane. The second segment turned perpendicular to the scanning plane ventrolateral to the right atrium, into the right atrioventricular groove. The third segment ran caudodorsally, almost perpendicular to the scanning plane, and continued into the right atrioventricular groove on the diaphragmatic surface of the heart.

### Diameter and length of the LCA and RCA

**Figure 7:**
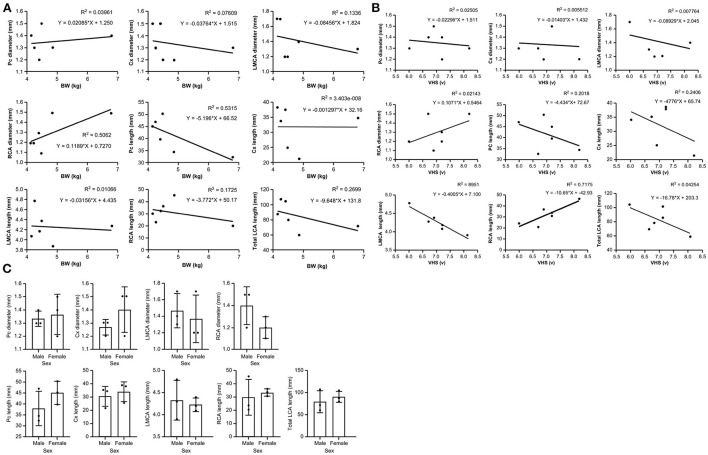
Scatter plots of the association between **(A)** body weight (BW) and major coronary branches and **(B)** vertebral heart score (VHS) and major coronary branches in domestic short-haired cats. **(C)** Box plots showing the correlation between sex and major coronary branches in domestic short-haired cats. Cx, circumflex; LCA, left coronary artery; LMCA, left main coronary artery; Pc, paraconal interventricular; RCA, right coronary artery.

Diameters and lengths of the LCA and RCA in all cats are listed in Table 4. The mean ± SD of the major coronary branches, 95% CIs, and *P*-values of the analyses for all major CA branches, BW, sex, and VHS are also provided (Table 5; Figure 7). All datasets were normally distributed. For all CA branches, the length and diameter exhibited no significant correlations with BW or sex (*P* > 0.05) (Table 5; Figure 7). We observed a significant negative correlation between VHS and LMCA, as well as a positive correlation between VHS and RCA (*P* < 0.05) (Table 5; Figure 7).

**Table 4:** Diameter and length of major coronary arterial branches.

**Case No**.	**Diameter (mm)**					**Length (mm)**					
	**LMCA**	**Pc**	**Cx**	**S**	**RCA**	**LMCA**	**Pc**	**Cx**	**S**	**Total LCA**	**RCA**
1	1.7	1.4	1.5		1.2	4.1	45.1	38.7		87.9	31
2	1.7	1.3	1.3	1.2	1.2	4.8	46.9	34.2	21.8	107.7	24
3	1.2	1.2	1.5	1.1	1.3	4.2	39.7	37.9	22.8	104.6	33.6
4	1.2	1.5	1.2		1.1	4.4	50.3	25.3		80	36.7
5	1.3	1.4	1.3		1.5	4.3	32.4	35.2		71.9	20.7
6	1.4	1.3	1.2		1.5	3.9	34.5	21.6		60	46

Cx, circumflex; LCA, left coronary artery; LMCA, left main coronary artery; Pc, paraconal interventricular; RCA, right coronary artery; S, septal.

**Table 5:** Correlation of body weight (BW) and vertebral heart score (VHS) with diameter and length of the left and right coronary arteries.

		**Mean ± SD**	**BW**		**VHS**	
			**95% CI**	***P*-values**	**95% CI**	***P*-values**
LMCA	Diameter	1.42 ± 0.23	−0.3836–0.2144	0.4762	−0.5165–0.3379	0.5929
	Length	4.28 ± 0.31	−0.4536–0.3905	0.8457	−0.5909– −0.2102	0.0043
Pc	Diameter	1.35 ± 0.1	−0.1217–0.1634	0.7054	−0.2218–0.1759	0.7646
	Length	41.48 ± 7.14	−11.97–1.576	0.1002	−16.68–7.809	0.3715
Cx	Diameter	1.33 ± 0.14	−0.2197–0.1444	0.5967	−0.2757–0.2476	0.8888
	Length	32.15 ± 7.04	−9.760–9.758	0.9997	−16.55–7.002	0.3232
Total LCA	Length	94.5 ± 17.08	−31.68–12.38	0.2909	−43.84–10.29	0.1604
RCA	Diameter	1.3 ± 0.17	−0.04412–0.2819	0.1128	−0.1777–0.3920	0.3552
	Length	32 ± 9.09	−15.24–7.696	0.4128	1.374–19.93	0.0333

CI, Confidence Interval; Cx, circumflex; LCA, left coronary artery; LMCA, left main coronary artery; Pc, paraconal interventricular; RCA, right coronary artery; SD, standard deviation.

## Discussion

The current findings demonstrate that ECG-gated MDCT angiography is an effective method for obtaining diagnostic images for the evaluation of feline CAs, without the need for heart rate modulation or beta-blocker administration. The scanning protocol used herein is the first to set the delay time for ECG-gated scans to non-ECG-gated images without bolus tracking or the use of a test bolus, thereby permitting the successful acquisition of ECG-gated scans. Although the scanning method was performed with the aim of directly comparing non-ECG and ECG-gated images in the same patient, our findings indicate that selecting the delay time, reducing radiation exposure, and widening the fourth or fifth non-ECG scan range may be beneficial for patients that require abdominal or whole-body scans beyond the heart. Therefore, the scanning method used herein may be clinically useful in veterinary medicine.

The detailed course of the CAs was difficult to evaluate on non-ECG MDCT scans due to poor opacification and severe motion artifacts, although the bilateral coronary ostium and proximal CA segments could be visualized on the second and third scans. These findings indicate that non-ECG MDCT scans can provide the minimal information required to evaluate congenital single CA diseases, similar to findings observed in canines (4, 11, 13).

This study is the first to describe feline LCA branching types using cardiac MDCT. Type II, which does not involve S branches, was the most predominant type, occurring in four of the six cats. This type has not been reported in dogs to date (5, 16, 18). In a previous morphological study of CAs in 48 cats, type I (49%) was the most predominant, followed by type II (26%). Here, we observed both left (four cats) and right (two cats) coronary dominance. These results highlight the morphological variability of feline CAs, consistent with a previous report (8). In one cat with type III branching (i.e., three main CA branches simultaneously extending from the LMCA), we observed severe tortuosity of the Pc and two additional efferent branches, a pattern that has not been reported in previous studies. Studies of humans have reported that coronary tortuosity may lead to alterations in coronary flow, resulting in a reduction in coronary perfusion pressure distal to the tortuous coronary arterial segment, subsequently leading to myocardial ischemia (19–21). Moreover, coronary tortuosity is positively associated with arterial hypertension and the female sex (19). The cat with coronary tortuosity herein was a 3.6-year-old intact male that exhibited normal clinical findings on the basic health check-up. Therefore, this cat should be monitored for myocardial disease with consistent cardiac assessments. Further research is warranted to clarify the clinical implications of feline coronary tortuosity.

In the current study, the diameters of the LCA and RCA were considered short relative to their length. The diameters of the LMCA and RCA were 1.42 ± 0.23 and 1.3 ± 0.17, respectively, in contrast to a previous report indicating that the diameter of the RCA was slightly larger (9). We also observed that three cats had a slightly larger RCA than LCA at 0.1–0.2 mm, in contrast to the previous finding indicating that the value for the LCA ostium was greater than that for the RCA (9). Considering the possibility of subtle measurement differences on CT images and the limited sample size in this study, our findings suggest the possibilitythat the LCA and RCA diameters may be similar. A canine study reported positive correlations between the CA and BW, including the LCA and RCA diameter, as well as the length of the Cx and Pc branches (16). However, no significant correlation between the diameter or length of CAs and BW was observed herein. This may have been due to the small sample size and small weight variation (4.82 ± 1.0 kg) of the cats in our study when compared with those in previous canine studies. The heaviest cat (Case 5) in our study had a similar CA diameter but shorter CA length than those of other cats, indicating that BW may not affect CA length or diameter.

Coronary dominance of the heart refers to whether the LCA or RCA perfuses the majority of the myocardial tissue. Variable methods have been adopted to determine this parameter (16, 17). For a comparison with canine coronary MDCT results, we investigated which CA supplied the subsinosal interventricular branch and which CA extended beyond the crux of the heart, as reported previously (16). Other parameters, including the origin or layout of the arteries at the heart apex, as well as the relative lengths and numbers of LCA/RCA branches, appeared similar between the present and previous studies. In contrast to the left-dominant coronary circulation in dogs, our findings indicate that cats exhibit variable dominance, despite the small sample size (8, 16, 17). Herein, the Cx branch of four left-dominant cats and the RCA branch of two right-dominant cats supplied the subsinosal interventricular branch with the extension beyond the crux cordis.

In the current study, the cat diagnosed with HCM during a basic health check-up exhibited a positive result on pro-BNP tests, cardiomegaly (VHS 8.2 v) on thoracic radiography, and regional thickening of the interventricular septum (0.66 cm) in the right parasternal short-axis view on ECG. As there were no abnormalities in the left atrium-to-aorta ratio (1.56; reference range, 0.88–1.7), diastolic function, systolic anterior motion of the mitral valve, or other examinations, the cat was able to undergo a cardiac CT scan. The cat with HCM exhibited a type II LCA branching pattern, which was the most predominant in our study, as well as right coronary dominance and marked right coronary length. This may have partly underpinned the positive correlation between VHS and RCA herein. HCM is a primary myocardial disorder, indicating that myocardial abnormalities are due to a defect, most often within the sarcomeres (i.e., individual contractile elements within the heart) of the cardiomyocytes, and are not secondary to other causes, such as hyperthyroidism, systemic hypertension, aortic stenosis, or acromegaly (22). We hypothesize that these contractile myocardial defects influence the contractile wall of the left heart with a diminished left coronary flow, resulting in compensatory concentric hypertrophy. This may have clinical implications for the association between CAs and pathophysiology in cardiomyopathic feline patients, such as those with HCM.

This study has some limitations, including a small sample size that comprised only five healthy cats and one with HCM. Further, we did not obtain confirmation based on gross findings or biopsy. Despite these limitations, our prospective study of feline CAs using MDCT holds clinical significance for veterinary medicine. In human medicine, the assessment of CA morphology is performed not only to diagnose certain diseases but also before open and endovascular or cardiovascular procedures (9). Furthermore, in veterinary medicine, anatomical studies of the aorta and CAs are conducted in animals and used as experimental models prior to human clinical trials (9). Although the surgical interventions mentioned above have not been widely performed in veterinary medicine, research in this field and increased awareness of pet owners may warrant such procedures. Detailed morphological knowledge of feline coronary vessels will enable novel diagnostic and therapeutic methods, as well as facilitate the implementation of endovascular procedures that are commonly used in humans in veterinary medicine. These approaches will be particularly beneficial for elucidating the pathophysiology of feline CA diseases and other heart diseases.

In conclusion, the cardiac MDCT scanning protocol, comprising non-ECG followed by ECG-gated MDCT scans, enabled adequate visualization of CAs without bolus tracking and provided useful information concerning feline CAs. These findings suggest that cats may potentially exhibit varieties of normal CA patterns. Further studies in a big number of healthy and cardiomyopathic cats are warranted to clarify the clinical feasibility of cardiac MDCT in the evaluation of feline CAs.

## Data availability statement

The original contributions presented in the study are included in the article/supplementary material, further inquiries can be directed to the corresponding author.

## Ethics statement

The animal study was reviewed and approved by Institutional Animal Care and Use Committee of Seoul National University (approval number: SNU-220113-4). Written informed consent was obtained from the owners for the participation of their animals in this study.

## Author contributions

JK: setting the direction and design for the overall study and writing the main paper. D-HK: writing the thesis, analyzing and interpreting data, and reviewing the patients' conditions. KK: collecting and analyzing the patients' data and contributing to the direction of this study. DO: analyzing data and considering clinical aspects of the study. JY: coordinating the overall flow and direction of the study and writing and editing the paper. All authors contributed to the article and approved the submitted version.

## Conflict of interest

The authors declare that the research was conducted in the absence of any commercial or financial relationships that could be construed as a potential conflict of interest.

## Publisher's note

All claims expressed in this article are solely those of the authors and do not necessarily represent those of their affiliated organizations, or those of the publisher, the editors and the reviewers. Any product that may be evaluated in this article, or claim that may be made by its manufacturer, is not guaranteed or endorsed by the publisher.

## Abbreviations

BW: body weight
CAs: coronary arteries
CI: confidence interval
Cx: circumflex
ECG: electrocardiography
HCM: hypertrophic cardiomyopathy
LCA: left coronary artery
LMCA: left main coronary artery
MDCT: Multidetector computed tomographic
NT-proBNP: N-terminal Pro-B-type natriuretic peptide
Pc: paraconal
RCA: right coronary artery
S: septal
SD: standard deviation
VHS: Vertebral Heart Score.
